# Simultaneous Bilateral Femoral Osteonecrosis in Gaucher Disease

**DOI:** 10.3390/life13051135

**Published:** 2023-05-06

**Authors:** Daniel Cohen, Yadin Levy, Yaron Bar-Ziv, Shoshana Revel-Vilk, Ari Zimran, Ehud Lebel

**Affiliations:** 1 Faculty of Medicine, The Hebrew University, Jerusalem 9112102, Israel; dannyc@szmc.org.il (D.C.); yadinl@szmc.org.il (Y.L.); zimran@md.huji.ac.il (A.Z.); 2 Department of Orthopedic-Surgery, Shaare Zedek Medical Center, Jerusalem 9103102, Israel; 3 Assaf Harofeh Medical Center, Tel Aviv 6997801, Israel; 4 Gaucher Clinic, Shaare Zedek Medical Center, Jerusalem 9103102, Israel

**Keywords:** Gaucher disease, osteonecrosis, total-hip-arthroplasty (THA)

## Abstract

Gaucher disease (GD) is one of the most common lysosomal storage disorders. Bone complications are the most critical irreversible consequence of GD. Osteonecrosis (ON) of the femoral head inevitably leads to osteoarthritis and may be managed by hip arthroplasty. The introduction and worldwide use of therapeutic agents (specifically enzyme replacement therapies (ERT)) lowered the prevalence of osteonecrosis events per patient. We present the cases of two female patients who sustained simultaneous bilateral femoral head osteonecrosis after receiving ERT for long periods while exposed to concomitant risk factors related to femoral head ON. Both patients suffered severe pain and deterioration of their daily activity capabilities, and thus, were offered bilateral hip arthroplasty. Surgery was performed in both hip joints during the same procedure. The current report highlights several key aspects of femoral head ON in young patients with GD.

## 1. Introduction

Gaucher disease (GD) is one of the most common lysosomal storage disorders. The enzyme dysfunction leads to the accumulation of non-degraded glucocerebroside in the lysosomes of macrophages [1]. Bone complications are the most critical irreversible consequence of GD; these complications include bone marrow infiltration, which may result in bone infarction; episodes of bone crises; osteonecrosis, mainly of large joints; and (less commonly) pathological fractures. Asymptomatic patients may present only distal metaphyseal femur widening (“Erlenmeyer flask deformity”), while more severely affected patients may sustain multiple bone osteonecrotic regions and, eventually, deformity and osteoarthritis in numerous joints [2]. Osteonecrosis (ON) of the femoral head in GD is detrimental. It almost inevitably leads to changes in the sphericity of the cartilaginous cover, and eventually leads to osteoarthritis. This condition commonly occurs during early adolescence, thus necessitating hip arthroplasty at a relatively young age [3]. Bilateral ON was reported in patients before enzyme-replacement therapy (ERT), and patients were managed through sequential hip arthroplasties (as required based on their symptoms). Bilateral ON is highly uncommon today as more severely affected patients receive treatment. Simultaneous bilateral ON of femoral heads in non-GD patients is also a rare event reported during pregnancy or after trauma [4,5]. It has not been previously reported in patients with GD. We present two female patients who developed simultaneous bilateral ON during childbearing age. Both patients received long-term ERT before the event. Due to severe hip joint pain and limitations in daily activities, both patients were offered hip arthroplasties.

## 2. Case Presentations

**Figure 1:**
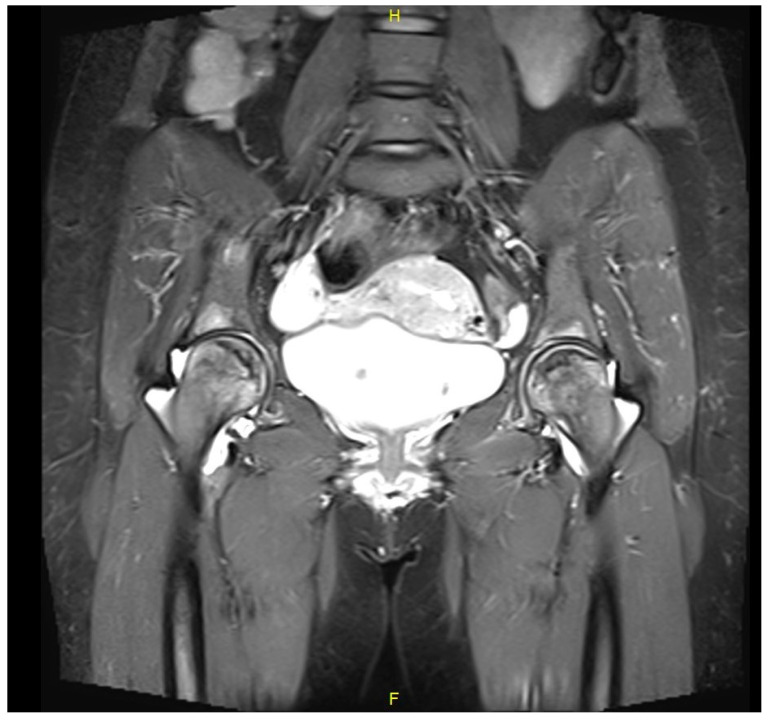
A coronal plane t-2 weighted MRI of pelvis and hip joints of Case 1 showing bilateral pre-collapse changes of femoral heads and joint effusions. These findings are compatible with simultaneous bilateral osteonecrosis.

**Figure 2:**
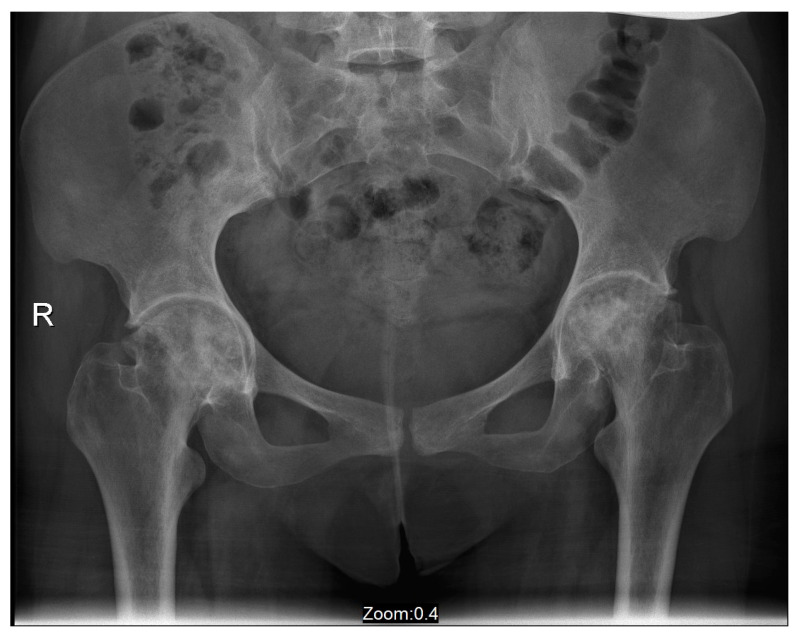
An antero-posterior X-ray of pelvis and hip joints of Case 1 performed four years after the acute events. These findings present severe bilateral osteoarthritis.

Case 1: A 39-year-old female was diagnosed with GD at age 12 following an investigation for abdominal pain and hepatosplenomegaly. The diagnosis was based on reduced enzyme activity and homozygosity for the N370S variant in the GBA1 gene (c.1226A > G). She also had a diagnosis of Celiac disease. She underwent a splenectomy at age 19 due to an enlarged “wandering” spleen. She started low-dose ERT (imiglucerase, 15 units/kg/2 weeks) at the age of 12, but stopped for personal reasons after six years (at age 18). At the age of 28, five months after the delivery of her third child and having not received ERT for approximately ten years, she complained of right groin pain. After two weeks, the pain increased and became permanent, and gradually the left groin also became painful. At the time, she had anemia (Hemoglobin 10.4 g/dL), and her chitotriosidase was elevated (3400 nm/mL/h). A magnetic resonance imaging (MRI) scan of her hip joints revealed bilateral changes in the femoral heads which were compatible with GD-related ON (Figure 1). ERT was immediately resumed (velaglucerase 60 units/kg/2 weeks). It was suggested that she try hyper-baric oxygen treatment to preserve the sphericity of the femoral heads and delay osteoarthritis concomitantly; she also undertook a range of motion exercises and limited ambulation as much as possible. After four years of non-operative support, both hip joints were severely damaged (Figure 2). She eventually underwent bilateral simultaneous total hip arthroplasty (via an anterior approach). Her rehabilitation after surgery was uneventful. Currently, she is pain-free and has unlimited ambulation.

**Figure 3:**
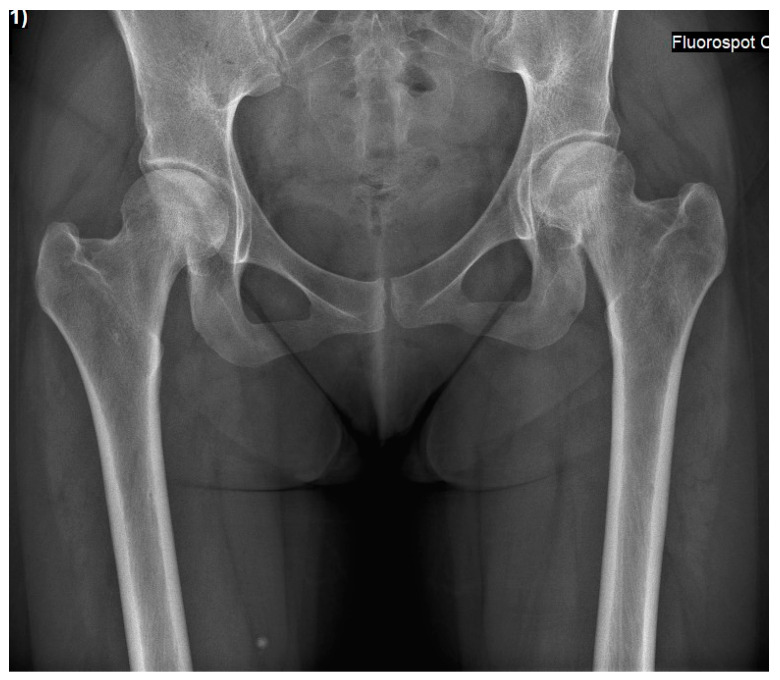
An antero-posterior pelvis X-ray of Case 2 was performed a few days after pain started, and it was already bilateral. These findings present a pre-collapse radiographic stage, where both femoral heads are spherical, and joint space is preserved.

**Figure 4:**
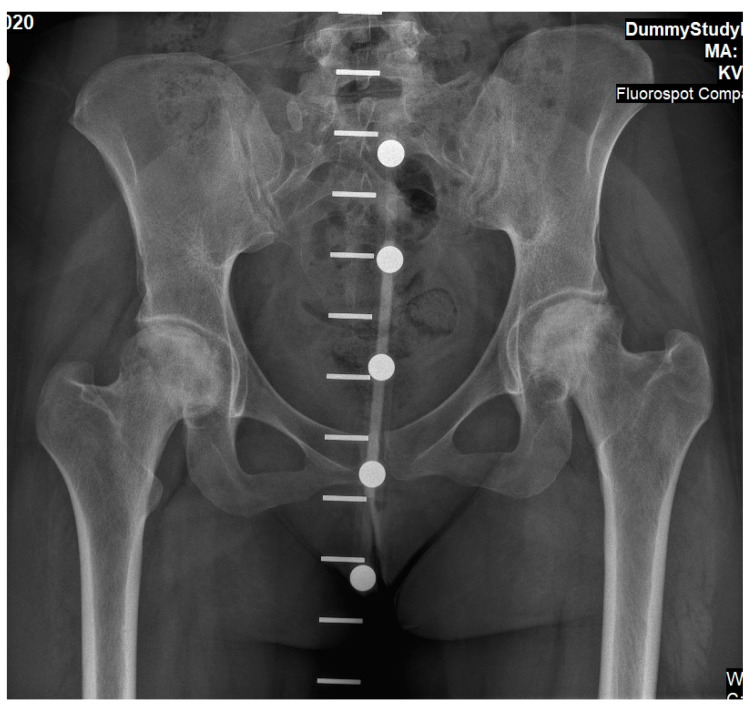
An antero-posterior X-ray of pelvis and hip joints of Case 2. This was taken as part of pre-operative planning for simultaneous bilateral hip arthroplasty (note the calibration marks used for implant selection). At this stage she was in severe pain. These findings present osteonecrosis changes to femoral heads, although sphericity is partially preserved and joint space is still evident.

**Figure 5:**
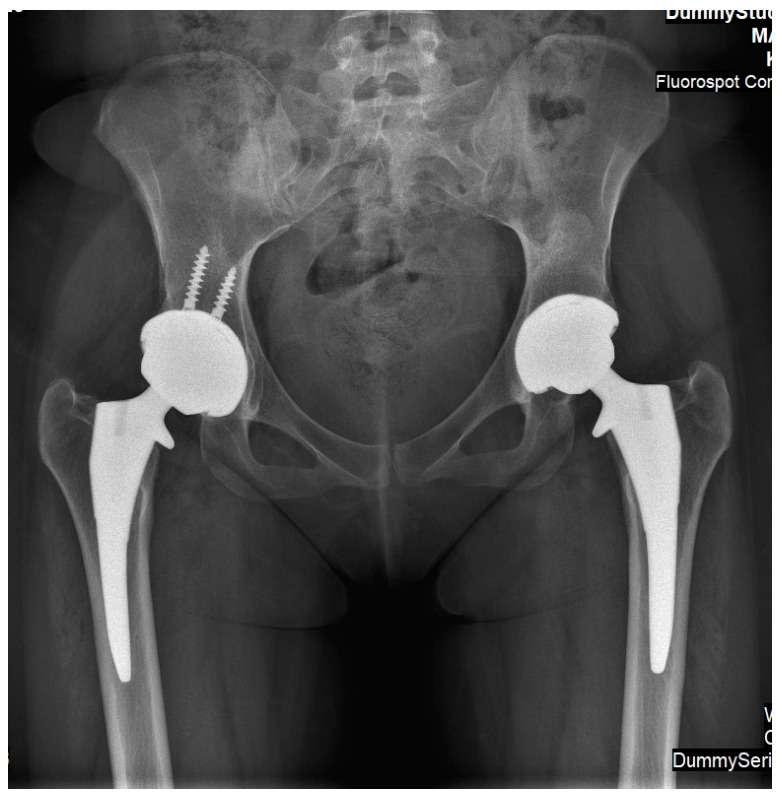
An antero-posterior X-ray of pelvis and hip joints of Case 2. It was performed after she underwent simultaneous bilateral hip arthroplasty. Implants are similar; however, acetabular cup on right side was augmented using two screws (based on surgeons’ preferences).

Case 2: A 27-year-old female was diagnosed with GD at age four following an investigation for hepatosplenomegaly, anemia, and thrombocytopenia. The diagnosis was based on reduced enzymatic activity and compound heterozygosity with the N370S/84GG variants in the GBA1 gene. She started receiving ERT shortly after her diagnosis (imiglucerase 60 units/kg/2 weeks), with an outstanding response (normalization of the hematological parameters, reduction in organomegaly and growth, etc.), and never skipped infusions. Her ERT dosage was lowered (imiglucerase 30 units/kg/2 weeks) at age 18 due to her stable status. At the age of 25, 10 months after the delivery of her third child, and while taking progestin-only oral contraceptives (and her regular ERT of velaglucerase 30 units/kg/2 weeks), she reported an insidious left hip pain unrelated to strenuous effort or trauma. Within a few days, the pain also involved the right hip. Radiographic changes in femoral heads were not evident (Figure 3), but MRI revealed early signs of bilateral pre-collapse femoral head osteonecrosis. It should be noted that her glucosylphingosine (lyso-Gb1) blood levels were always around 50 ng/mL until 6 months before the acute event, when the level increased to 162 ng/mL. She underwent 60 sessions of hyper-baric oxygen treatment without improvement. She gradually developed chronic pain and limited motion in both hip joints with concomitant radiographic arthritic changes (Figure 4). Due to painful bilateral osteoarthritis, she underwent simultaneous bilateral total hip arthroplasties (anterior approach, Figure 5). Histological evaluation of femoral heads that were resected during surgery confirmed extensive bilateral osteonecrosis. Her post-surgery rehabilitation was uneventful, and she is currently pain-free and has no limitations in her daily activities.

## 3. Discussion

The current cases represent a very uncommon occurrence of simultaneous bilateral femoral head osteonecrosis in two patients with GD. In both instances, osteonecrosis was diagnosed in early stages (before the femoral heads were showing radiographic changes, Ficat stage I). In both cases, an MRI confirmed that the clinical findings indeed represented new-onset cases of simultaneous–bilateral osteonecrosis. The course of the disease and eventual management were similar: Both patients developed osteoarthritis and underwent bilateral total hip arthroplasty in one setting.

The etiology of femoral head ON is not fully understood, although thrombosis of arterial supply to the femoral head is probable. This assumption is based on the similarity of this process to the consequences of an inadvertent surgical ligation of epiphyseal arteries. Other (systemic) etiologies may cause necrosis of the femoral head. These events are occasionally bilateral and may involve other bones (multifocal osteonecrosis). Bilateral (non-simultaneous) involvement was reported in sickle-cell disease [6,7], Legg–Calve–Perthes disease [8], corticosteroid treatment, and alcohol abuse [9]. It is possible that the pathology causing ON is similar. An explanation of bilateral affection is the “multiple-event theory,” assuming that the involvement of both sides during the same period of time is due to repeated (or continuous) insults (i.e., thrombosis), eventually leading to bilateral bone changes. The present cases demonstrate a very uncommon simultaneous occurrence. This rare occurrence warrants an investigation of the uniqueness of these two patients’ conditions and how they lead to these detrimental events.

As noted from the detailed history of the patients described above, both had very similar courses. Both patients were of similar age and gender, and each had a risk of hyper-coagulability (pregnancy and oral contraceptives). A possible supporting factor was the interrupted use of ERT (Case 1). We had previously diagnosed femoral head ON in patients having their ERT paused for a few months. The patient presented in Case 2 was the only one known to us who sustained femoral head osteonecrosis while receiving ERT uninterruptedly. Nevertheless, the doses of ERT used were lower than those given in other centers; this event may be attributed to an insufficient dosage. GD is not considered a hyper-coagulation state. Thus, ERT discontinuation may not explain the tendency for arterial thrombosis. Increased inflammatory status was described in GD over two decades ago [10,11,12]. Inflammatory vascular disease of bone is an etiological theory that should be evaluated. Inflammation may lead to thrombosis in a specific hyper-coagulation period. ON is a sub-acute painful event that slowly worsens. Within a few days (up to a few months) patients report severe pain, limitation of joint range motion, and reduced ambulation. Bone biopsies performed early (if core–decompression is performed) or late (during THA surgery) reveal bone necrosis; however, an arterial thrombosis was not described in tissue specimens. Heretofore, it was not proven to be a vascular infarct or an inflammatory vasculitis [13]. Other theories are fatty material embolism and vascular compression due to bone marrow engorgement by Gaucher cells [14,15]. These theories were also not supported by histologic findings, although infiltration of femoral heads by lipid laden Gaucher cells is described [13].

Bone involvement, specifically ON of large peri-articular regions of bone, was more prevalent before ERT was available. One explanation for this decline is the impact of ERT on the systemic severity of the disease, including the bones. The second is the elimination of splenectomy. In the pre-ERT era, splenectomy was suggested for severely affected patients. Eventually, splenectomized patients suffered from rapidly progressive bone involvement due to increased infiltration of their bone marrow. Analysis of data from the international Gaucher Register (ICGG) of Genzyme/Sanofi clearly marked the previous splenectomy as the independent most predictive risk factor for development of ON, aside from timing of therapy initiation [16,17]. Both patients in the current study had an increased risk of ON during the specific period of their event. Case 1 underwent a splenectomy at an early age and received ERT irregularly. Case 2 had a more severe disease (related to her genetic background). Despite receiving un-interrupted ERT since early childhood (and having intact spleen), her pregnancy and later use of progestin oral contraceptives probably increased the risk of ON. While the low-dose protocol cannot be excluded as contributing to the skeletal complication reported in Case 2, the rarity of such cases does not allow a clear conclusion related to this long-term controversy. Moreover, the low levels of the most specific and sensitive biomarker (Lyso-Gb1) may support the claim that it was probably another trigger, such as the hyper-coagulability during pregnancy or hormonal therapy, that contributed to ON. The unexpected rise in the Lyso-Gb1 level [18] in Case 2 should have been perceived as a warning sign of the development of osteonecrosis, which is an additional take home-message of this report.

Before ERT was available, there was a reluctance regarding performance of THA in patients with GD who suffered from osteoarthritis of the hip joint. Most important was the patient’s general condition: patients suffering from debilitating skeletal problems were usually those with more severe visceral diseases (specifically those with multiple spleen infarctions). Patients’ ambulatory capacity was limited, and the benefit from THA was lowered. The survival of patients with GD was mistakenly presumed to be significantly shortened. Therefore, such a surgical intervention was not suggested. This surgical procedure is not risk-free, especially for patients with hematologic disorders that may lead to coagulopathy and chronic anemia. The risk of infection is higher in GD patients, especially those who underwent splenectomy. Necrosis of the femoral head, indicating hip-arthroplasty for GD patients, was thought to have less favorable outcomes than other osteonecrosis etiologies [19]. Finally, the ability of bone to integrate into the femoral and acetabular metallic components of the prosthetic implant was uncertain. Osteoporosis is common in patients with Gaucher disease and may expose patients to intra-operative fractures or loosening of the implant. A few case series of patients undergoing THA were reported during the pre-ERT era [20,21,22]. These studies advocated that selected patients with hip osteoarthritis may benefit from this procedure with reasonable risks. Patients were pre-conditioned through correcting their anemia and thrombocytopenia, and experienced orthopedic consultants of GD clinics performed the surgery. In 1994, the systemic treatment of GD made a significant leap with the introduction of ERT [23]. This treatment decelerated the gradual worsening condition of these patients, allowing for improved general and surgical outcomes [24]. In the post-ERT era, the discussion regarding THA for patients with GD became entirely different. Patients were now seeking improvement in quality of life. Thus, proper timing and the best performance of this procedure were its goals. Postponing THA until older age was claimed to be beneficial; previous studies revealed inferior results in young Gaucher patients undergoing hip replacement due to their higher lifestyle demands [25]. On the other hand, improved longevity due to implant design improvement could reduce the disability of younger patients, enhancing their quality of life and function [26,27]. Non-Gaucher patients sustaining bilateral hip disease may be offered a simultaneous bilateral hip replacement. Although this is a lengthier procedure, patients are spared doubled rehabilitation periods. Unlike idiopathic osteoarthritis, a Gaucher patient with significant hematological abnormalities might have higher anesthetic and surgical risks during bilateral THA than during one-sided surgery [28]. Features regarding the innovative approach of simultaneous bilateral hip-arthroplasty are beyond the scope of this report. This report suggests that such an approach is viable even for patients with GD. For the current two patients, the Gaucher Clinic’s consultants were confident that those young patients could safely undergo simultaneous bilateral THA.

## 4. Conclusions

The current report highlights several aspects of femoral head ON in young patients with GD. The first aspect is ERT’s beneficial effect and its discontinuation’s detrimental effect. The second aspect is the effect of ERT dose, splenectomy, pregnancy, and hypercoagulability state on the risk of ON. The third aspect is the ability to safely perform hip arthroplasty in patients with GD, even in the form of a simultaneous bilateral procedure.
